# Selenium-Based *S*-Adenosylmethionine Analog Reveals the Mammalian Seven-Beta-Strand Methyltransferase METTL10 to Be an EF1A1 Lysine Methyltransferase

**DOI:** 10.1371/journal.pone.0105394

**Published:** 2014-08-21

**Authors:** Tadahiro Shimazu, Joaquin Barjau, Yoshihiro Sohtome, Mikiko Sodeoka, Yoichi Shinkai

**Affiliations:** 1 Cellular Memory Laboratory, RIKEN, Wako, Japan; 2 Synthetic Organic Chemistry Laboratory, RIKEN, Wako, Japan; Universität Stuttgart, Germany

## Abstract

Lysine methylation has been extensively studied in histones, where it has been shown to provide specific epigenetic marks for the regulation of gene expression; however, the molecular mechanism and physiological function of lysine methylation in proteins other than histones remains to be fully addressed. To better understand the substrate diversity of lysine methylation, *S*-adenosylmethionine (SAM) derivatives with alkyne-moieties have been synthesized. A selenium-based SAM analog, propargylic Se-adenosyl-l-selenomethionine (ProSeAM), has a wide spectrum of reactivity against various lysine methyltransferases (KMTs) with sufficient stability to support enzymatic reactions *in vitro*. By using ProSeAM as a chemical probe for lysine methylation, we identified substrates for two seven-beta-strand KMTs, METTL21A and METTL10, on a proteomic scale in mammalian cells. METTL21A has been characterized as a heat shock protein (HSP)-70 methyltransferase. Mammalian METTL10 remains functionally uncharacterized, although its ortholog in yeast, See1, has been shown to methylate the translation elongation factor eEF1A. By using ProSeAM-mediated alkylation followed by purification and quantitative MS analysis, we confirmed that METTL21A labels HSP70 family proteins. Furthermore, we demonstrated that METTL10 also methylates the eukaryotic elongation factor EF1A1 in mammalian cells. Subsequent biochemical characterization revealed that METTL10 specifically trimethylates EF1A1 at lysine 318 and that siRNA-mediated knockdown of METTL10 decreases EF1A1 methylation levels *in vivo*. Thus, our study emphasizes the utility of the synthetic cofactor ProSeAM as a chemical probe for the identification of non-histone substrates of KMTs.

## Introduction

Protein post-translational modification (PTM) is involved in diverse cellular processes such as gene expression, signal transduction, and intracellular interactions. Lysine methylation in histones has been extensively studied for more than a decade and has various important roles in gene expression, replication and cell division, and genome stability [1]–[3]. Although hundreds of non-histone lysine methylation sites have been reported, the enzymes responsible for these lysine methylations and their physiological functions remain largely unknown. Several approaches have been developed to probe specific lysine-methylated proteins. One such method is to utilize antibodies specific to methylated lysine [4], [5]. A second approach takes advantage of methylated lysine binding domains. It has been reported that an engineered version of the triple malignant-brain-tumor domain region of L3MBTL1 can serve as a probe for the detection and enrichment of proteins containing mono- and di-methylated lysine residue(s) [6], [7]. Another approach is to use cofactor analogs for labeling, detection, and pull-down via copper-catalyzed azide-alkyne cycloaddition (CuAAC), also known as click chemistry to enrich PTM targets. Studies on synthetic cofactors for lysine methylation have been reported for several SET domain lysine methyltransferases (KMTs) such as SUV39H2 [8], G9a/GLP [8], [9], ESET [10], and Set7/9 [9] (also reviewed in [11]). Similar approaches have been attempted on other PTMs such as glycosylation [12], acetylation [13], and lipidation [14]–[16].

The human genome encodes over 200 methyltransferases (MTases), including 50 SET-domain-containing KMTs [17]. It has been reported that some SET domain KMTs methylate not only histones but also non-histone proteins [18]. For example, the proteins G9a and SMYD2 are known to methylate the tumor suppressor p53 [19], [20]. It has been reported that SMYD2 also methylates a chaperone protein, Hsp90 [21], whereas SETD1A methylates another chaperone protein, Hsp70 [22]. More recently, the substrate diversity of non–SET domain KMTs has emerged as a subject of interest. VCP has been shown to be methylated by a seven-beta-strand KMT, METTL21D [23], whereas Hsp70 is methylated by another seven-beta-strand KMT, METTL21A [24], [25]. Moreover, calmodulin has been reported to be methylated by a non-SET domain KMT, calmodulin-lysine N-methyltransferase (CamKMT) [26]. Mammalian METTL10 remains uncharacterized, whereas the yeast METTL10 ortholog See1 has been shown to methylate yeast elongation factor eEF1A [27], [28].

In the present report, a selenium-based *S*-adenosylmethionine analog, propargylic Se-adenosyl-l-selenomethionine (ProSeAM), was synthesized as a probe for the identification of non-histone substrate(s) of KMTs. ProSeAM has been reported as a useful probe for several KMTs, but so far it has been mainly used to characterize SET domain KMTs and Prmt arginine methyltransferases (RMTs) [8], [9]. We established a substrate identification method useful for both SET and non-SET domain KMTs, using target enrichment via chemical probes and subsequent quantitative MS identification. With this method, we identified substrates for two non-SET domain KMTs, METTL21A and METTL10, in mammalian cells on a proteomic scale. Finally, we show that EF1A1 is responsible for tri-methylation of lysine 318 of EF1A1 *in vivo*.

## Materials and Methods

### Synthesis of propargylic Se-adenosyl-l-selenomethionine

Synthesis of propargylic Se-adenosyl-l-selenomethionine (ProSeAM) was performed as described in [8] with a few modifications. Briefly, Se-adenosyl-l-homoselenocysteine and propargyl bromide (60 equiv.) were reacted in formic acid in the presence of silver trifluoromethanesulfonate for 20 h at room temperature (RT). After the reaction, compounds were purified with HPLC, and their structures were confirmed by ^1^H NMR.

### Antibodies

Antibodies used were obtained as follows: anti-α-tubulin antibody (clone B-5-1-2, Sigma-Aldrich, St. Louis, MO, USA); anti-HSP70 antibody (clone C92F3A-5, Millipore, MA, USA); anti-FLAG M2 antibody (Sigma-Aldrich).

### Plasmids

Plasmids for GST-G9a [29] and FLAG-ESET [30] were obtained as described in the literature. Full-length cDNA corresponding to human HSPA1A/HSP70 [22], human HSP90, human EF1A1, human METTL21A (NCBI ID: NM_145280), mouse SMYD2 (NCBI ID: AK145261), and mouse METTL10 (NCBI ID: AK008476) were obtained by PCR from a template plasmid, as a clone from the HeLa cDNA library, or as a clone from the Fantom3 clone collection [31]. The cDNAs obtained were cloned into the pET19b vector to generate N-terminal His-tagged constructs (pET19b-METTL21A, pET19b-SMYD2, pET19b-METTL10), N-terminal His-2× FLAG-tagged construct (pET19b-HFF-HSP90) or cloned into the pDEST17 vector to generate N-terminal His-tagged construct (pDEST17-HSP70). The cDNAs were also cloned into the pcDNA3 vector with a C-terminal FLAG-tag (pcDNA3-cFLAG) or into a modified pCAG vector containing a N-terminal 3× FLAG-tag and an IRES-Puromycin cassette (pCAGFIP) to generate mammalian expression constructs (pcDNA3-METTL10-cFLAG, pCAGFIP-EF1A1). The QuikChange Site-Directed Mutagenesis Kit (Agilent Technologies, Inc., Santa Clara, CA, USA) was used to generate mutant plasmids.

### Purification of recombinant proteins

*Escherichia coli* BL21 (pLysS) strains were transformed with pET19b plasmids, and the bacteria were cultured in 2× YT medium with ampicillin (100 µg/mL) and 0.5 mM isopropyl β-d-1-thiogalactopyranoside (IPTG) for 2 h at 37°C. The cells were pelleted and lysed with 1× PBS/0.5% NP-40 by sonication with a Branson Sonifier (S-250D, Branson Ultrasonics Corp., CT, USA) for 5 min on ice. The lysates were centrifuged at 15,000×*g* for 10 min, and the supernatants were incubated with Ni-NTA Agarose (Qiagen, Valencia, CA, USA) for 1 h at 4°C with gentle agitation. The agarose beads were washed 5 times with wash buffer (50 mM Tris-HCl, pH 7.4, 25 mM imidazole) and then eluted with elution buffer (50 mM Tris-HCl pH 7.4, 250 mM imidazole). The purified proteins were dialyzed with 1× PBS/10% glycerol, and the concentration was measured using the Bradford Protein Assay Kit (BioRad Laboratories, Hercules, CA, USA).

### MALDI-MS analysis of a histone H3 peptide

An N-terminal histone H3 peptide (20 µM) corresponding to amino acids 1–21 (ARTKQTARKSTGGKAPRKQLA) was incubated for 2 h at 20°C with cofactors (400 µM) with or without purified GST-G9a (2 µM) [29]. The reacted peptide was analyzed with Microflex LT (Bruker Daltonics Inc., MA, USA).

### Labeling of full-length substrates with ProSeAM

One microgram of recombinant full-length histone H3, His-HSP90 or His-HSP70 was incubated in 1× reaction buffer (50 mM Tris-HCl, pH 8.0) with GST-G9a (0.5 µg), FLAG-tagged KMTs (0.5 µg), His-SMYD2 (1 µg) or His-METTL21A (1 µg) with or without ProSeAM (250 µM, unless otherwise noted) for 2 h at 20°C. The reaction was stopped by the addition of four volumes of ice-cold acetone. The reaction tube was centrifuged at 15,000×*g* for 5 min, and precipitates were washed once with ice-cold acetone. The pellet was resolved in 15.5 µL 1×PBS+0.2% SDS; then, 4 µL of 5× click reaction buffer (7.5 mM sodium ascorbate, 0.5 mM Tris-(benzyltriazolylmethyl) amine (TBTA), 5 mM CuSO_4_) and 0.5 µL of 10 mM Azide-PEG4-Biotin (Click Chemistry Tools, Scottsdale, AZ, USA) were added, and the mixture was incubated for 60 min at RT to allow the CuAAC reaction to proceed [10]. The reaction was stopped with four volumes of ice-cold acetone, and the pellet was resolved in Laemmli SDS-sample buffer. Proteins were separated on a 12.5% or 15% acrylamide gel and transferred to a nitrocellulose membrane (Pall Corporation, Port Washington, NY, USA); the membrane was incubated with streptavidin-HRP (Thermo Fisher Scientific Inc., Waltham, MA, USA) for 1 h at RT. The membrane was washed three times with 1×PBS, incubated with the Western Lightning Plus-ECL Kit (Perkin Elmer, Waltham MA, USA) according to the manufacturer's protocol, and the chemiluminescence was detected with X-ray film (RX-U, FUJI-FILM, Minato-ku, Tokyo, Japan).

### *In vitro* labeling of mammalian cell lysates with ProSeAM and KMTs

One hundred and fifty micrograms of HEK293T cell lysates in lysis buffer (50 mM Tris-HCl, pH 8.0, 50 mM KCl, 10% glycerol, 0.1% Tween-20) were incubated with 400 µM ProSeAM and 15 µg of recombinant KMT in 1× reaction buffer (50 mM Tris-HCl, pH 8.0, total reaction volume was 75 µL) for 2 h at 20°C. The reaction was stopped by adding four volumes of ice-cold acetone. The reaction tube was centrifuged at 15,000×*g* for 5 min, and precipitates were washed once with ice-cold acetone. The pellet was resolved in 58.5 µL of 1× PBS +0.2% SDS, then 15 µL of 5× click reaction buffer and 1.5 µL of 10 mM Azide-PEG4-Biotin (Click Chemistry Tools) were added; the reaction was then incubated for 60 min at RT. The click reaction was stopped with four volumes of ice-cold acetone. The pellet was resolved in 75 µL of binding buffer (1× PBS, 0.1% Tween-20, 2% SDS, 20 mM DTT) and sonicated for 10 s. One and a half milligrams of Dynabeads M-280 Streptavidin (Life Technologies Japan Ltd., Minato-ku, Tokyo, Japan) in 225 µL of IP buffer (TBS, 0.1% Tween-20) were added and incubated for 30 min at RT (the final SDS concentration in the reaction was 0.5%). The protein-bound beads were washed 3 times with wash buffer (1× PBS, 0.1% Tween-20, 0.5% SDS) and then washed twice more with 100 mM ammonium bicarbonate (ABC) buffer. The protein-bound beads were analyzed by western blot or mass spectrometry.

### Quantitative MS/MS analysis for target protein identification

Acetonitrile (1/10 volume) and DTT (20 mM) were added to the protein-bound Dynabeads in 100 mM ABC buffer, and the mixture was incubated for 30 min at 56°C. Then, iodoacetamide (IAA) was added and the mixture was incubated for 30 min at 37°C in the dark. The protein samples were then digested with 0.5 µg trypsin (Promega). The trypsinized protein fragments were applied to a liquid chromatograph (EASY-nLC 1000; Thermo Fisher Scientific, Odense, Denmark) coupled to a Q Exactive Hybrid Quadrupole-Orbitrap Mass Spectrometer (Thermo Fisher Scientific, Inc., San Jose, CA, USA) with a nanospray ion source in positive mode. The peptides derived from the protein fragments were separated on a NANO-HPLC capillary column C18 (0.075-mm inner diameter ×150 mm length, 3 mm particle size; Nikkyo Technos, Tokyo, Japan). Mobile phase “A” was comprised of water with 0.1% formic acid, and mobile phase “B” was comprised of acetonitrile with 0.1% formic acid. Two different slopes were used for a 60 min gradient at a flow rate of 300 nL/min: 5%–35% B in 48 min and then 35%–65% B in 12 min. The mass spectrometer was operated in the top-10 data-dependent scan mode. The parameters of the mass spectrometer were as follows: spray voltage, 2.3 kV; capillary temperature, 275°C; mass-to-charge ratio, 350–1800; normalized collision energy, 28%. Raw data was acquired with the Xcalibur software (Thermo Fisher Scientific). The MS and MS/MS data were searched against the Swiss-Prot database using Proteome Discoverer 1.4 (Thermo Fisher Scientific) with the MASCOT search engine software, version 2.4.1 (Matrix Science, London, United Kingdom). The search parameters were as follows: enzyme, trypsin; static modifications, carbamidomethyl (Cys); dynamic modifications, oxidation (Met); precursor mass tolerance, ±6 ppm; fragment mass tolerance, ±20 mDa; maximum missed cleavages, 1. The proteins were considered identified when their false discovery rates (FDR) were less than 1%. The obtained data were further filtered as follows: peptide rank, 1 and peptides per proteins, minimal 3. Nonlabel quantification was performed by Top3-TIC method with Proteome Discoverer. Briefly, peptide areas of three most abundant peptides were used for quantification of protein amounts. For substrate identification, proteins with at least a 2-fold increase in three independent experiments were defined as positive hit proteins.

### *In vitro* methylation assay with 14C -labeled SAM

One microgram of FLAG-tagged EF1A1 were incubated in 1× Collin's buffer (25 mM Tris-HCl pH 8.5, 5 mM DTT) with His-METTL10 (1 µg) and 14C-labeled SAM (0.01 µCi, Perkin Elmer) for 2 h to overnight at 30°C. The reaction was stopped by adding Laemmli SDS-sample buffer. Proteins were resolved on a 12.5% acrylamide SDS-PAGE gel, and the dry gel was exposed to an imaging plate (FUJI-FILM) for 48 h, and the autoradiography was detected using a BAS-5000 Image analyzer (FUJI-FILM).

### Identification of methylation sites by MS/MS analysis with CD3-SAM

One microgram of FLAG-tagged EF1A1 were incubated in 1× Collin's buffer with His-METTL10 (1 µg) and CD3-SAM (200 µM) for 3 h at 30°C. The reaction was stopped by adding Laemmli SDS-sample buffer. Proteins were resolved on a 12.5% acrylamide SDS-PAGE gel, excised from the gel, and digested with the endoproteinase Asp-N. The peptide fragments were analyzed using a Q Exactive Hybrid Quadrupole-Orbitrap Mass Spectrometer (Thermo Fisher Scientific) as described above, except the following parameters: dynamic modifications, Oxidation (M), Methyl(CD3) (K), Dimethyl(CD3) (K), Dimethyl(CD3)(CH3) (K), Trimethyl(CD3) (K), Trimethyl(CD3)2(CH3)1 (K), Trimethyl(CD3)1(CH3)2 (K).

### Immuno fluorescence microscopy

Plasmids for FLAG-tagged METTL10 were transfected into HeLa cells grown on coverslips using Lipofectamine2000 (Life Technologies) according to the manufacturer's protocol. Twenty four hours after transfection, the cells were washed with PBS and fixed with 3.7% HCHO/PBS for 10 min at RT. Fixed cells were permeabilized with 0.5% TritonX-100/PBS for 10 min and blocked with 5% bovine serum albumin in PBS for 30 min. Fixed cells were incubated with primary antibodies for 1 h and then stained with Alexa Fluor-conjugated secondary antibodies (Alexa Fluor 488 Goat Anti-Mouse IgG (H+L) Antibody, Life Technologies). Fluorescent images were obtained using a DeltaVision microscope (Cornes Technologies, Minato-ku, Tokyo, Japan).

### siRNA knockdown experiment

siRNA reagents against human METTL10 were obtained from Thermo Fischer Scientific (Dharmacon, ON-TARGETplus Human METTL10 siRNA). HEK293T cells were transfected with either scramble siRNAs or METTL10 siRNA with DharmaFECT 1 Transfection Reagent (Thermo Fischer Scientific) according to the manufacturer's protocol. Twenty-four hours after siRNA transfection, cells were transfected with pCAGFIP-EF1A1 and cultured for another 48 hours. Cells were harvested and pelleted in two tubes (for RT-PCR and quantification of methylated peptides).

### RT-PCR

siRNA treated cells were lysed in Sepasol-RNA I Super G (nacalai tesque, Kyoto, Japan) and total RNA were collected according to the manufacture's protocol. cDNA were synthesized with Omniscript RT Kit (Qiagen) using random 9-mer primer. RT-PCR was performed with StepOnePlus Real-Time PCR Systems (Life Technologies). The primers used were as follows: hMETTL10_Q_F TGGACCAAGGAAGAGTTGCT; hMETTL10_Q_R GGAGGCTGAAGTGGAAAAGA; hGAPDH-F TGCACCACCAACTGCTTAGC; hGAPDH-R GGCATGGACTGTGGTCATGAG.

### Quantification of methylated EF1A1 peptides

siRNA treated cells were lysed in 1× PBS/0.5% NP-40/0.2 mM PMSF, and the FLAG-EF1A1 was immunoprecipitated with M2 agarose (Sigma-Aldrich). Methylation of EF1A1 was analyzed by in-gel-digestion with the endoproteinase Asp-N. The peptide fragments were analyzed using the triple stage quadrupole mass spectrometer TSQ Vantage EMR (Thermo Fischer Scientific) for quantification of methylated and unmethylated EF1A1 peptides. Peptide intensity was normalized to the peaks of the following peptide fragments of EF1A1: 220-DGNASGTTLLEAL-232; 243-DKPLRLPLQ-251; 389-DGPKFLKSG-397; 417-DYPPLGRFAVR-427; and 428-DMRQTVAVGVIKAV-441.

## Results

### ProSeAM functions as a cofactor for SET domain KMTs and seven-beta-strand KMTs

**Figure 1:**
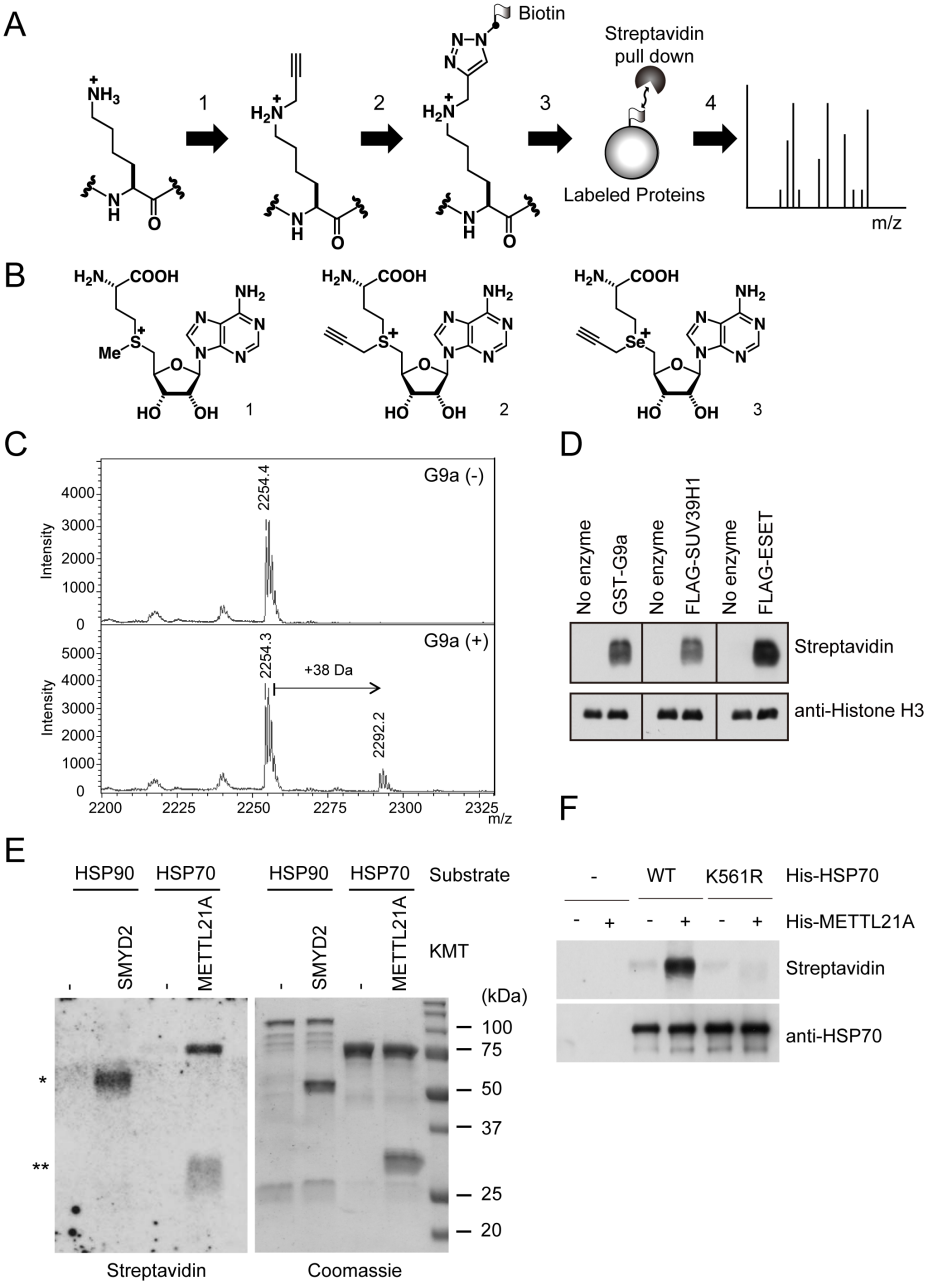
ProSeAM, a synthetic SAM analog, has a wide spectrum of reactivity for histones and non-histone substrates. A, Schematic overview for analyzing lysine methylation. A synthetic cofactor was used to transfer an alkyne moiety to the ε-amino group of lysine by KMTs (1). The modified proteins were tagged with biotin via CuAAC reaction (2). Tagged-proteins in the crude lysates were pulled down with affinity beads (3), and the precipitants were further analyzed with a LC-MS apparatus (4). B, Chemical structure of SAM (1), propargylated SAM (2) and ProSeAM (3). C, H3 peptide (1-21 a.a.) and ProSeAM was incubated with or without GST-G9a at 20°C for 2 h, then the peptide was analyzed by MALDI-TOF MS. D, full-length Histone H3 (1 µg) and ProSeAM (500 µM) were incubated with indicated KMTs (0.5 µg) for 2 h at 20°C. The histones were separated by SDS-PAGE, transferred to a nitrocellulose membrane and probed with streptavidin-HRP (top) or anti-Histone H3 antibody (bottom). E, The non-histone substrates His-HSP90 and His-HSP70 (1 µg) were incubated with His-SMYD2 and His-METTL21A (1 µg), respectively. After the reaction, proteins were separated by SDS-PAGE (right). Their modifications were detected by western blotting with streptavidin-HRP as in Fig. 1D. *and ** showed automodification of SMYD2 and METTL21A, respectively (left). F, His-HSP70 (WT and K561R) were incubated with or without His-METTL21A in the presence of ProSeAM for 2 h at 20°C. Modified proteins were biotinylated and detected with streptavidin-HRP (top) or anti-HSP70 antibody for the loading control (bottom).

Histone lysine methylation by SET domain containing KMTs such as G9a, ESET, and Suv39H1 is well studied, whereas non-histone lysine methylation has yet to be addressed. To identify substrates for SET domain KMTs as well as seven-beta-strand KMTs, a click-chemistry based strategy was employed (Fig. 1A). SAM derivatives, which contained alkyne-moieties were synthesized, and their stability and reactivity against various wild-type KMTs were evaluated. Propargyl-SAM (Fig. 1B2) was very unstable and unable to detect modified peptides or full-length histones *in vitro* (not shown). This result is consistent with previous reports that the half-life of Propargyl-SAM was very short in the reaction buffer (pH 8.0) [8], [9]. However, ProSeAM (Fig. 1B3) has been reported as a cofactor for a series of SET domain KMTs (GLP/G9a, SUV39H2, PRSET7/9) as well as other MTases (PrmC, PRMT1) [8], [9]. We also tested the stability and ability to transfer the propargyl group to histone peptides or full-length histone H3, and the modifications by GST-G9a were confirmed by both MALDI-MS (Fig. 1C) and western blot with streptavidin-HRP after the propargyl moiety was conjugated with a biotin tag via the CuAAC reaction (Fig. 1D). In addition, we confirmed that the side chain of ProSeAM could be transferred to histones by a series of SET domain KMTs (G9a, ESET, SUV39H1) (Fig. 1D). Next, we tested if non-histone KMTs could transfer the methyl analog propargyl group to their non-histone substrates (Fig. 1E). SMYD2 has been shown to methylate HSP90 [32], whereas a seven-beta-stand KMT METTL21A methylates HSP70 family proteins [24], [25]. Recombinant His-SMYD2 and His-METTL21A were incubated with His-HSP90 and His-HSP70, respectively. Although SMYD2 auto-modification was observed (Fig. 1E asterisk), no apparent modification was detected on HSP90 (Fig. 1E left panel). On the other hand, a significant modification on HSP70 by METTL21A was observed (Fig. 1E left panel, ∼75 kDa band). To assess the reaction specificity of METTL21A with ProSeAM, the known methylation site of HSP70 [22], [24], [25] was mutated to arginine (HSP70-K561R), and the modification behavior of the mutant was investigated (Fig. 1F). HSP70-K561R showed only background level modification, suggesting that METTL21A transfers the propargyl-group to lysine 561, the known methylation site. These results clearly indicate that ProSeAM can function as a cofactor with certain non-histone KMTs including seven-beta-strand KMTs.

### Proteomic identification of substrates labeled with ProSeAM by KMTs

**Figure 2:**
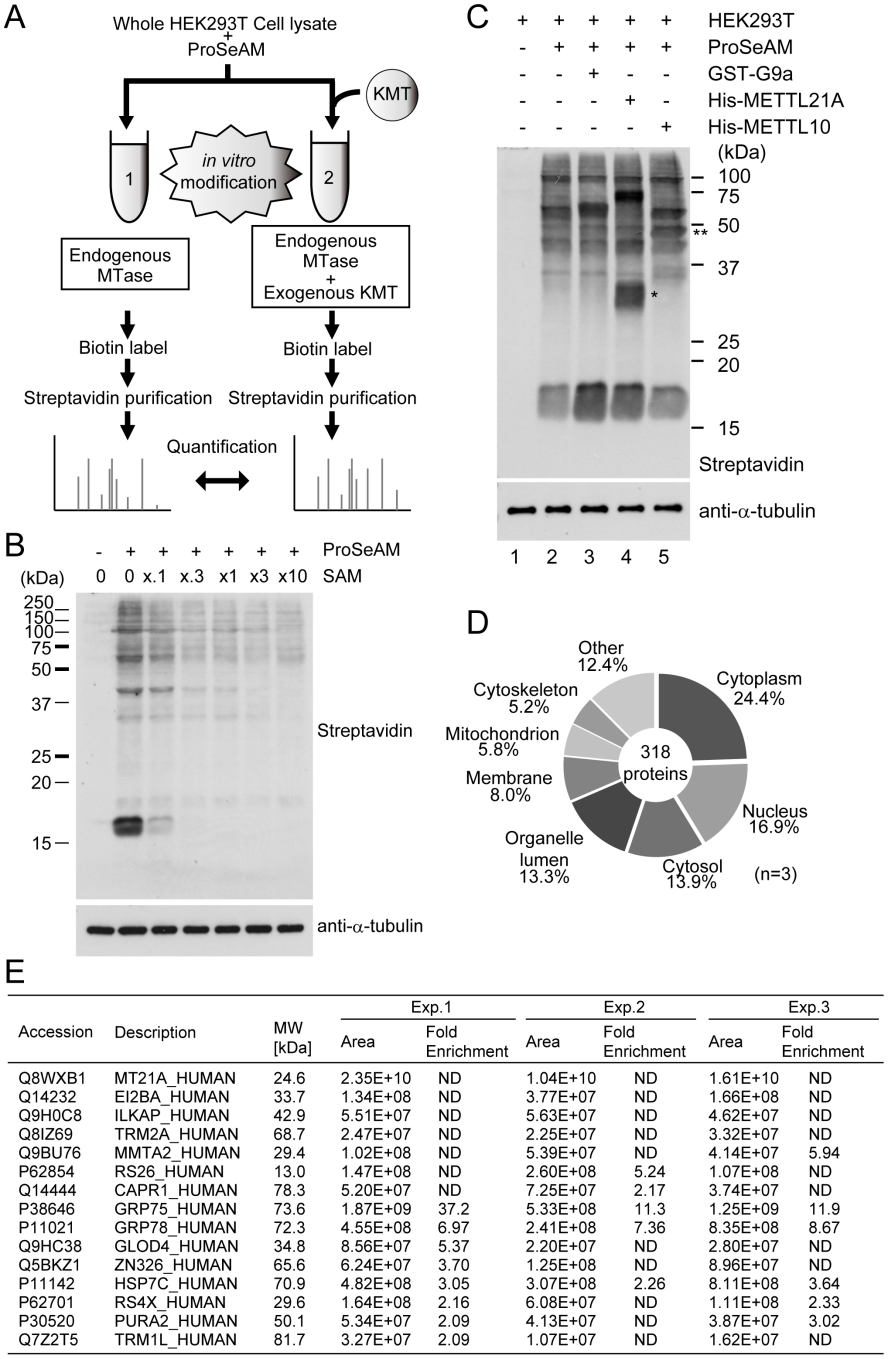
Proteomic identification of substrates for seven-beta-strand MTases. A, Schematic protocol for proteomic identification. HEK293T cell lysates were added to either propargylic Se-adenosyl-l-selenomethionine (ProSeAM) alone (1) or ProSeAM plus recombinant KMT (lysate:enzyme ratio was 10∶1) (2). After the *in vitro* reaction, labeled proteins were tagged with biotin and then precipitated with streptavidin beads. The precipitants were then digested with trypsin, and the trypsinized protein fragments were analyzed by LC-MS/MS. B, ProSeAM competes with SAM in the labeling reaction. HEK293T cell lysates were incubated with ProSeAM (250 µM) in the presence or absence of the indicated amount of SAM (0 to 2.5 mM). Modified proteins were biotinylated and detected with streptavidin-HRP (top). Equal protein loading was confirmed by western blotting with anti-α-tubulin antibody (bottom). C, western blot of labeled proteins. A 5% input of precipitated proteins without ProSeAM (1), with ProSeAM alone (2), with ProSeAM plus GST-G9a (3), with ProSeAM plus His-METTL21A (4) or with ProSeAM plus His-METTL10 was separately analyzed with western blotting with streptavidin-HRP (top) prior to the MS analysis, to compare the labeled proteins. Equal protein loading was confirmed by western blotting with anti-α-tubulin antibody (bottom). D, Doughnut chart of the subcellular distribution of proteins labeled with ProSeAM. HEK293T lysates alone (lane 1 in Fig. 2C) and HEK293T lysates with ProSeAM (lane 2 in Fig. 2C) were analyzed as described in A and Experimental procedures (n = 3). In total, 318 proteins were identified as ProSeAM-labeled proteins. E, List of METTL21A substrates. HEK293T cell lysates and ProSeAM were incubated with or without METTL21A (lane 2 and lane 4 in Fig. 2C), and analyzed as above. Molecular weight, peptide area (reflecting the quantity of detected protein), and fold enrichment of the peptide area are listed: ND, not determined because the substrate was detected only in the condition for lane 4 of B. The total numbers of identified proteins, 2-fold increase (compared to control in each experiment), and overlapped identified numbers of 3 independent experiments are listed in Table S2.

To identify protein substrates for a KMT of interest, we designed a screening method (Fig. 2A). Here, mammalian HEK293T cell lysates were incubated with ProSeAM and a specific KMT (1/10 volume weight of cell lysates). After *in vitro* modification, proteins were precipitated and resuspended in the click reaction buffer, and a biotin tag was conjugated to the alkyne moiety via a CuAAC reaction. Proteins were resuspended in IP buffer, and streptavidin beads were added to the reaction tubes to pull down biotin-labeled proteins. Quantitative comparisons of enriched proteins between endogenous MTases (Fig. 2A1) and endogenous MTases plus a KMT of interest (Fig. 2A2) were performed in the LC-MS/MS analysis, since there are endogenous MTases in the reaction mixture. To confirm these modifications with ProSeAM occurred via a MTase-dependent enzymatic reaction, an increased amount of SAM (up to 10-fold) was added in the HEK293T cell lysate alone incubation. As shown in Figure 2B, various bands were detected with Streptavidin-HRP after biotinylation via the CuAAC reaction and the majority of those signals were diminished by SAM addition in a dose-dependent manner, demonstrating that majority of the labeled molecules result from the SAM-dependent propargylation reaction.

Figure 2C shows a representative western blot with streptavidin-HRP after *in vitro* methylation with ProSeAM in the presence or absence of the indicated KMTs. As described, various proteins in HEK293T cell lysates were labeled with ProSeAM alone (Fig. 2C lane 1 vs. lane 2). Quantitative MS analysis revealed that 318 proteins were enriched more than 2-fold with ProSeAM treatment (Fig. 2D and Table. S1). The subcellular distributions of labeled proteins were not only restricted in the nucleus, but also exist in the cytosol and subcellular organelle. The robust detection of enriched proteins is likely due to the broad reactivity of ProSeAM against endogenous MTases in HEK293T cells, including KMTs and RMTs, a finding consistent with previous work [8]. When GST-G9a was added to the lysate, an increase in histone H3 modification, corresponding to a 17 kDa band, was observed (Fig. 2C; compare lane 2 vs. lane 3). Then, METTL21A was added to the lysate to test whether substrates for a seven-beta-strand KMT could be detected as well (Fig. 2C; lane 4). As expected, a significant increase in the intensity of the 70 kDa bands was observed compared to the control (lane 2 vs. lane 4). Moreover, a slight increase of the 17 kDa band was observed by METTL21A addition. To determine whether METTL21A labels histone H3 with ProSeAM *in vitro*, recombinant histone H3 was incubated with METTL21A in the presence of ProSeAM (Fig. S1). METTL21A clearly labeled histone H3, although the catalytic activity was much lesser than that of G9a. Subsequent quantitative MS analysis confirmed significant (more than 2-fold) enrichment of METTL21A targets (Fig. 2E) including METTL21A itself as automodification (Fig. 2C, asterisk) and HSP70 proteins (GRP75, GRP78, and HSP7C) which have been reported as METTL21A substrates [24], [25]. However, histone H3 was not defined as a hit protein due to its poor enrichment levels (the worst being 1.40-fold enrichment).

We considered the reproducibility of their enrichment values. Three independent quantitative MS comparison analyses were performed. 147, 139, and 52 proteins were defined as potential METTL21A substrates, respectively (Shown in Table S2). However, only 15 of these hits, including HSP70 proteins, were recognized as positive in all three experiments. We speculate that the low reproducibility of these experiments is caused by a high background signal of reference samples without exogenous KMT addition (Fig. 2C and 2D). Although the current protocol has some technical drawbacks, our results clearly show that the overall strategy works well for identification of known substrates for MEETL21A [24], [25].

### Mammalian METTL10 is an MTase

**Figure 3:**
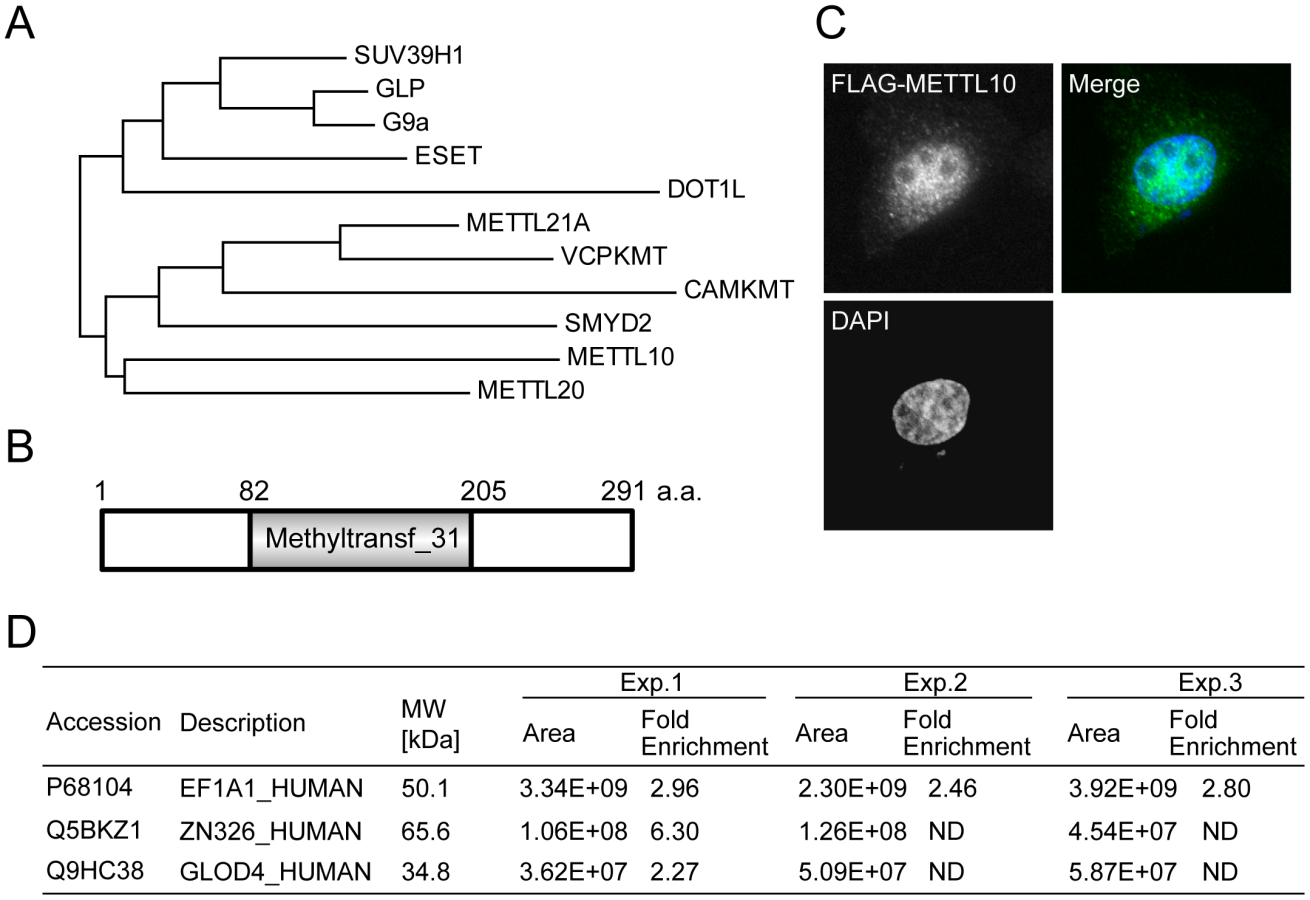
Mammalian METTL10 is an EF1A1 KMT. A, Phylogenic tree of human KMTs. Proteins were clustered based on DNA sequence by the maximum likelihood method using MEGA version 6 software [45]. The nucleotide sequences used here were as follows: G9a (NCBI ID; NM_006709), GLP (NCBI ID; NM_024757), SUV39H1 (NCBI ID; NM_001282166), ESET (NCBI ID; NM_001145415), DOT1L (NCBI ID; NM_032482), VCPKMT (NCBI ID; NM_024558), CAMKMT (NCBI ID; NM_024766), SMYD2 (NCBI ID; NM_020197), METTL21A (NCBI ID; NM_145280), METTL10 (NCBI ID; NM_212554), and METTL20 (NCBI ID; NM_001135863). B, Schematic structure of human METTL10. A conserved MTase domain, Methyltransf_31 (Pfam ID; PF13847) is located in the middle region. C, Subcellular localization of METTL10. The plasmid for FLAG-tagged METTL10 was transfected into HeLa cells, and the FLAG-tagged proteins were visualized under immunofluorescence microscopy. D, List of METTL10 substrates. HEK293T cell lysates and ProSeAM were incubated with or without METTL10 (lane 2 and lane 5 in Fig. 2C) and analyzed as in Figure 2. Molecular weight, peptide area which reflects the protein amount, and fold enrichment of the peptide area are listed. ND, not determined because substrate was detected only in the condition for lane 5 of Figure 2C. The total numbers of identified proteins, 2-fold increase (compared to the control in each experiment), and overlapped identified numbers of 3 independent experiments are listed in Table S3.

The human genome encodes over 200 MTases, including 50 SET-domain-containing KMTs, and many of these MTases remain uncharacterized [17]. METTL10 belongs to a seven-beta-strand MTase superfamily and contains the conserved MTase domain Methyltransf_31 (Pfam ID; PF13847) (Fig. 3A and B). Neither the MTase activity nor its cellular localization of mammalian METTL10 is known, although the yeast ortholog See1 has been shown to di-methylate lysine 316 on eEF1A [27], [28]. Therefore, we applied our method to study it.

First, we examined the cellular localization of METTL10. FLAG-tagged METTL10 was transiently expressed in HeLa cells, and its subcellular localization was examined using fluorescence microscopy (Fig. 3C). METTL10 was found to be localized to both the nucleus and the cytoplasm, suggesting that this protein may have cytoplasmic as well as nuclear function(s).

To detect the MTase activity of METTL10, we applied our ProSeAM assay. As shown in Fig 2C, a significant increase in the intensity of ∼50 kDa band was detected in samples incubated with METTL10 (lane 5, **), indicating that it possesses MTase activity and is capable of utilizing ProSeAM for the enzymatic reaction. To identify the METTL10 substrates in this assay, quantitative MS comparison analyses were performed in three times (Fig. 2B; lane 2 and lane 5) and three proteins, EF1A1 (UniProtKB ID: P68104), ZNF326 (UniProtKB ID: Q5BKZ1), and GLOD4 (UniProtKB ID: Q9HC38), were determined to be potential substrates (Fig. 3D and Table. S3).

### METTL10 trimethylates lysine 318 in EF1A1

**Figure 4:**
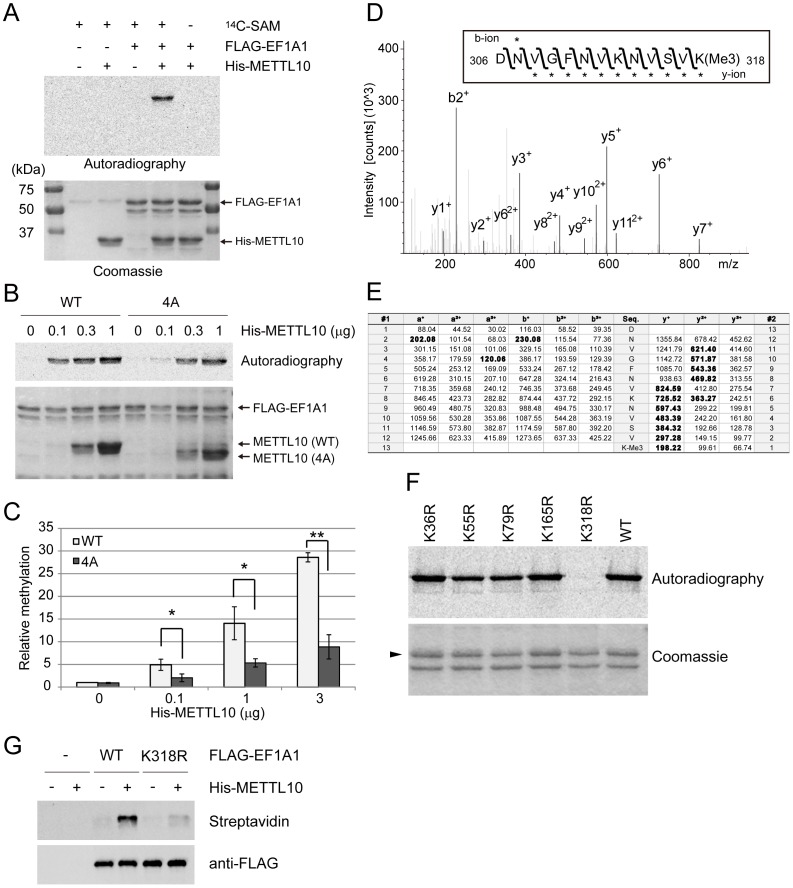
METTL10 tri-methylates K318 in EF1A1. A, METTL10 methylates EF1A1 *in vitro*. FLAG-tagged EF1A1 and His-METTL10 were incubated with or without 14C-labeled SAM. Proteins were separated with SDS-PAGE and stained with Coomassie blue (bottom), the autoradiography was detected with the image analyzer BAS-5000 (top). B, A conserved SAM-binding domain is important for MTase activity of METTL10. The MTase domain of METTL10 (WT, 85-DIGTGNG-91) was replaced with alanines (4A, 85-AIATANA-91). The representative gel and its autoradiography was detected as in A. C, The relative EF1A1 methylation was quantified by the intensity of autoradiography, and normalized to the intensity by WT 0 µg as 1 (n = 3, mean ± SD, *p<0.05, **p<0.01). D, MS/MS spectrum of peptide fragments containing trimethylated lysine 318. Box; Asterisks represent b- and y- ions detected. E. Table of the peptide fragment corresponding to amino acids 306-318. Bold numbers represents fragment ions detected in the experiment. F, METTL10 specifically methylates lysine 318. Five lysine methylation sites (lysine 36, lysine 55, lysine 79, lysine 165 and lysine 318) on EF1A1 were substituted with arginine, and their methylations were examined by autoradiography. G, FLAG-EF1A1 (WT and K318R) were incubated with or without His-METTL10 in the presence of ProSeAM for 2 h at 20°C. Modified proteins were biotinylated and detected with streptavidin-HRP (top) or anti-FLAG antibody for loading control (bottom).

A subsequent *in vitro* radioisotope labeling experiment with ^14^C-SAM was conducted to test if the three potential substrates determined in the above step were indeed methylated by METTL10. As shown in Fig. 4A, FLAG-tagged EF1A1 purified from HEK293T cells was clearly methylated by METTL10 *in vitro*. On the other hand, we could not confirm that METTL10 methylates either ZNF326 or GLOD4 (data not shown).

We further characterized the mechanism of methylation of EF1A1 by METTL10. Since METTL10 possesses a conserved SAM-binding motif, DxGxGxG, four amino acids in this motif (WT, 85-DIGTGNG-91) were substituted with alanine (4A, 85-AIATANA-91) to generate a SAM-binding deficient mutant. An *in vitro* methylation assay using ^14^C-SAM showed that the 4A mutant displays significantly reduced methylation activity toward EF1A1 (Fig. 4B and 4C), confirming that the methylation of EF1A1 by METTL10 is MTase-domain dependent.

To determine the methylation site(s) of EF1A1 by METTL10, an LC-MS/MS assay was performed. Here, FLAG-EF1A1 was modified *in vitro* by His-METTL10 in the presence of CD3-SAM, in which the reactive methyl group of SAM is replaced with CD3 in order to distinguish newly modified site(s) from endogenously methylated site(s). In this assay, newly modified site(s) by METTL10 are labeled with CD3-methyl, whereas endogenously methylated site(s) are labeled with CH3-methyl. The Asp-N digested peptide fragments were analyzed by LC-MS/MS. Figure 4D shows a fragmented peptide corresponding to amino acids 306–318 of EF1A1 that contains a tri-methylated lysine at position 318. Although b-ions were not well observed, the y-ions (y1 to y11) were detected after the fragmentation, which clearly demonstrated that lysine 318 is the tri-methylation site (Fig 4D and E).

It has been reported that five lysine methylation sites (lysine 36, lysine 55, lysine 79, lysine 165 and lysine 318) exist in mammalian EF1A1 [33], [34]. To further confirm the methylation site(s) on EF1A1 by METTL10, each reported lysine-methylated residue was substituted for arginine, and the methylation level was measured by autoradiography in the presence of His-METTL10 (Fig. 4F). The K318R mutant showed no methylation, whereas the other mutants had methylation levels similar to wild-type EF1A1. Next, FLAG-tagged wild-type EF1A1 (WT) or K318R were labeled with ProSeAM in the presence or absence of METTL10, and their modification were detected with Streptavidin-HRP after biotinylation via the CuAAC reaction. As shown in figure 4G, no apparent modification was detected at K318R, which further demonstrates that lysine 318 is the target site for METTL10. We conclude that METTL10 tri-methylates lysine 318 in EF1A1.

### Knockdown of METTL10 decreases the methylation of lysine 318 in EF1A1 *in vivo*

**Figure 5:**
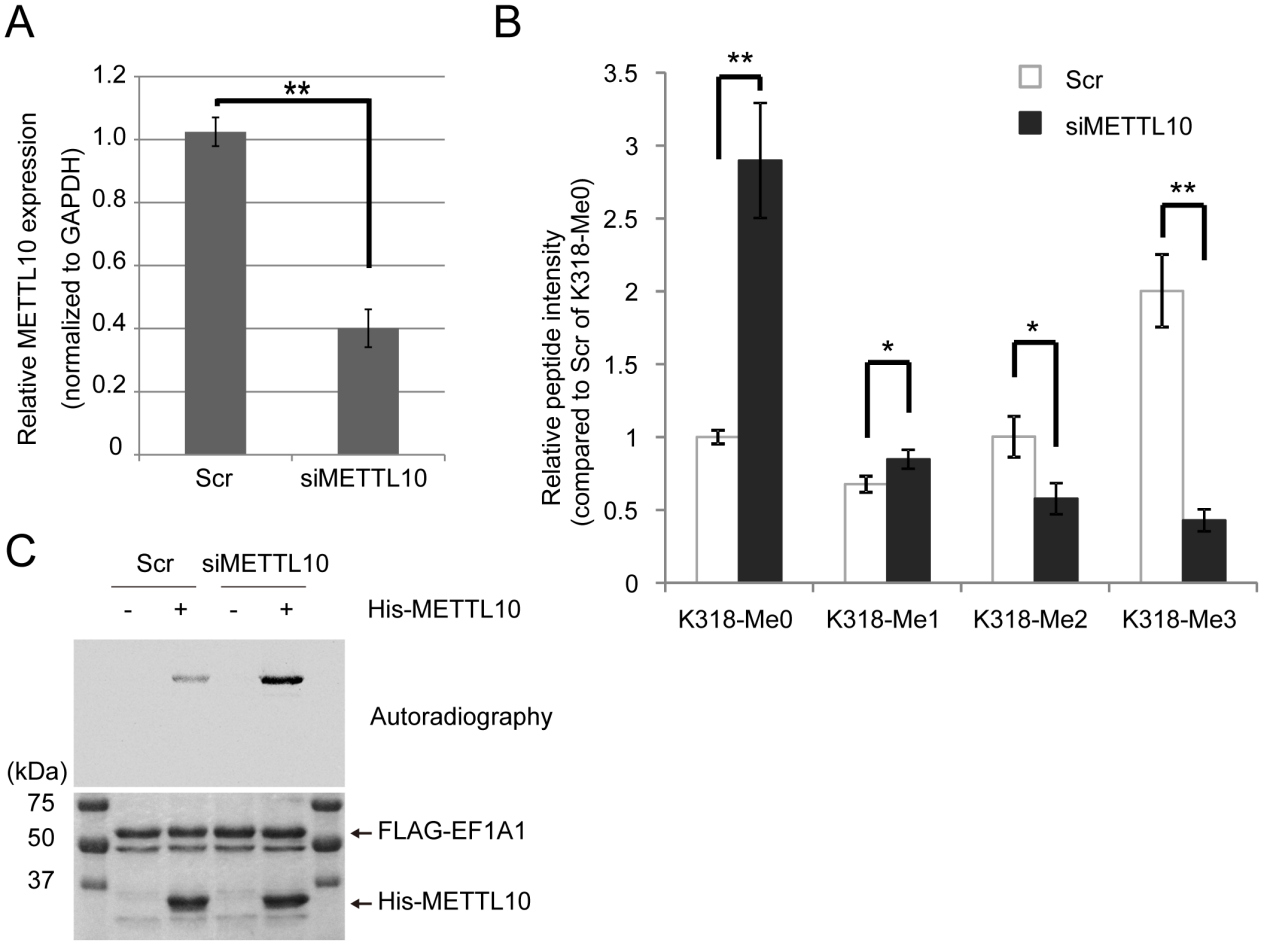
METTL10 knockdown reduces the level of lysine 318 methylation in EF1A1 *in vivo*. A, HEK293T cells were transfected with either scramble siRNA (Scr) or siRNA against METTL10 (siMETTL10). Twenty-four hours after the transfection, the plasmid for FLAG-EF1A1 was transfected and cultured for an additional 48 hours. Cells were harvested and the METTL10 expression level was analyzed with RT-PCR. n = 3, mean ± SD, **p<0.01. B, FLAG-EF1A1 was purified with anti-FLAG agarose beads from siRNA treated cells. EF1A1 lysine 318 mono-methylation (K318-Me1), di-methylation (K318-Me2), tri-methylation (K318-Me3), and unmethylation (K318-Me0) were analyzed by using a triple stage quadrupole mass spectrometer. The intensity of each peptide was normalized to the quantity of each sample's EF1A1 as determined by the presence of five EF1A1 peptide fragments, and the relative intensity was normalized to Scr of K318-Me0; n = 3, mean ± SD, *p<0.05, **p<0.01. C, *In vivo* METTL10 knockdown is required for *in vitro* robust methylation by METTL10. FLAG-EF1A1 purified from Scr or siMETTL10 treated cells was incubated with or without His-METTL10 in the presence of ^14^C-labeled SAM. Proteins were separated with SDS-PAGE and stained with Coomassie blue (bottom), the autoradiography was performed using a BAS-5000 image analyzer (top).

Finally, we examined if METTL10 is important for EF1A1 methylation *in vivo*. For this purpose, METTL10 was depleted from HEK293T cells with siRNA oligos. The mRNA expression of METTL10 was analyzed by RT-PCR (Fig. 5A). The level of EF1A1 methylated at lysine 318 in either scramble oligo- (Scr) or siRNA METTL10 oligo- (siMETTL10) transfected cells was measured by a mass spectrometer. As shown in Fig. 5B, METTL10 knockdown significantly reduced both di-methylation and tri-methylation of lysine 318 and increased the level of the un-methylated form of EF1A1 as compared to the Scr control. This result indicates METTL10 is also responsible for lysine 318 methylation *in vivo*.

To determine the state of methylation in METTL10 depleted cells, FLAG-EF1A1 from either Scr or siMETTL10 treated cells were methylated *in vitro* in the presence or absence of His-METTL10 with ^14^C-SAM. As shown in figure 5C, FLAG-EF1A1 from siMETTL10 cells were more methylated *in vitro*, which indicates low level methylation *in vivo*. From these results, we conclude that METTL10 is an important KMT for the methylation of lysine 318 in EF1A1 in HEK293T cells.

## Discussion

### ProSeAM as a chemical probe for lysine methylation with a wide spectrum of reactivity

To identify substrates for KMTs on a proteomic scale, we synthesized cofactors with an alkyne moiety. We tested the reactivity of several SAM analogs against various KMTs including both SET and non-SET enzymes (data not shown). Of these, we used the propalgylated, selenium-based *S*-adenosylmethionine analog ProSeAM (Fig. 1B), since the short half-life of propalgylated SAM made it hard to use as a probe for methylation, as mentioned in previous studies [8], [9]. ProSeAM has a propargyl group, which consists of an alkyne moiety and a reactive carbon, instead of a native methyl group. ProSeAM is the minimal engineered cofactor when compared to any other synthetic cofactors which have extended carbon chains in addition to an alkyne moiety [8], [9], [11]. This results in a wide spectrum of reactivity against native KMTs, which in turn enable us to screen substrates for certain KMTs without engineering their SAM-binding pockets (Fig. 1D and 1E). ProSeAM showed sufficient stability both under storage conditions (−80°C in 0.1% TFA, for 6 months) and reaction conditions (20°C, pH 8.0, for 1–2 h). The wide spectrum and stability of ProSeAM made it possible to be used as a chemical probe for various KMTs. Thus, ProSeAM has been reported as a catalytic probe of multiple protein methyltransferases including GLP, G9a and SUV39H2 (SET domain KMTs) as well as a cysteine methyltransferase NleE by Bothwell et al. [8]. Willnow et al. have also reported that ProSeAM is a versatile substrate with wild-type protein methyltransferases such as G9a, Set7/9 (SET domain KMTs), PRMT1 (RMT) and a bacterial glutamine MTase PrmC [9]. These two previous studies raise the question as to whether ProSeAM can be utilized for non-SET domain KMTs as well. In this report, we demonstrated that two seven-beta-strand MTases, METTL21A and METTL10 can utilize ProSeAM as a cofactor. We found that METTL21A labeled HSP70 family proteins (Fig. 2E), a finding consistent with previous studies [24], [25]. Moreover, our screening approach successfully determined the catalytic activity of METTL10 (Fig. 2C and 3). Subsequent proteomic identification revealed that EF1A1 is a substrate of METTL10 (Fig. 3D). However, ProSeAM is still an analog of SAM and so may be inert in the catalysis of certain KMTs. Indeed, ProSeAM was found to be inactive for HSP90 labeling by SMYD2 (Fig. 1E). Therefore, further synthesis and subsequent characterization of SAM analogs are also required in order to label substrates for such KMTs.

### The technical concerns of the current protocols

The use of quantitative MS analysis with ProSeAM made it possible to identify targets for METTL21A and METTL10. However, the screening strategy presented in this report has some drawbacks. As described above, a high background signal of the reference sample without any exogenous KMT addition is the major concern. In order to minimize the false-positive findings, it is necessary to repeat the quantitative MS analysis several times. If the background signal can be reduced, there may be a better chance to identify less abundant substrates. Inactivation or depletion of endogenous MTase activities before the *in vitro* enzymatic reaction with ProSeAM is one obvious way to decrease the background signal. Another way of decreasing the background signal is development and employment of target KMT specific cofactor(s) instead of using the wide spectrum cofactors such as ProSeAM. It does not improve the background issue, the use of SILAC, iTRAQ, or other relevant quantification methods may increase accuracy. As demonstrated for the METTL10-EF1A1 case (Figure 5C), depletion or reduction of the target KMT from the cells used for the substrate source can improve S/N ratio (i.e. fold enrichment) in MS experiments. This is particularly useful if the target proteins are already heavily methylated inside of the cells. Lastly, if cofactors that are suitable for *in vivo* labeling could be developed, experiments using them could greatly improve the understanding of the dynamics of protein lysine methylation during development or proliferation in living cells and animals.

### METTL10 is a mammalian EF1A1 methyltransferase which specifically trimethylates lysine 318

METTL10 is a seven-beta-strand MTase, which is conserved among various species from *Saccharomyces cerevisiae* to humans. Although the yeast METTL10 ortholog See1 is reported to di-methylate lysine 316 on eEF1A, it was unclear up until now as to whether METTL10 possesses intrinsic MTase activity. In the present report, we have shown that mammalian METTL10 has MTase activity and also target/methylates EF1A1 *in vivo*. Mammalian METTL10 tri-methylates lysine 318, whereas yeast See1 di-methylate the corresponding lysine 316. This finding suggests that an evolutionarily conserved regulatory mechanism of methylation exists in EF1A. It has been reported that EF1A contributes many cellular processes beyond the delivery of aminoacyl-tRNAs to the ribosome. These processes include unclear export of aminoacyl-tRNAs [35], ubiquitin-dependent protein degradation [36], organization of the actin cytoskeleton [37], and replication of several RNA viruses [38]-[41] (reviewed in [42]). Recently, Li et al. reported that methylation of eEF1A by yeast METTL10-like See1 facilitates RNA virus replication in yeast and plants [43]. More recently, Vermillion et al. showed that methylation of EF1A1 is required for neural crest migration in chicken embryos [44]. Although the biological significance of lysine 318 methylation in EF1A1 in mammals has yet to be addressed, it is possible that methylation of lysine 318 affects neural crest migration as well as RNA virus replication, given the fact that the methylation site is conserved among yeast, chicken, and human. Since knocking down METTL10 decreases both di-methylation and tri-methylation of EF1A1 *in vivo* (Fig. 5B), whereas yeast eEF1A does not exist in tri-methylated form, it could be intriguing to determine if di-methylation and tri-methylation functions differently between the various biological functions of EF1A1.

## Supporting Information

Figure S1 **Histone H3 is labeled by METTL21A with ProSeAM.** Full-length Histone H3 (1 µg) and ProSeAM (500 µM) were incubated with indicated KMTs (0.5 µg) for 2 h at 20°C. The histones were separated by SDS-PAGE, transferred to a nitrocellulose membrane and probed with streptavidin-HRP (top) or anti-Histone H3 antibody (bottom).
(TIF)

Table S1 Proteins labeled with ProSeAM by endogenous MTase. ND; Not determined because protein was detected only in the presence of ProSeAM.
(XLS)

Table S2 Summary of proteomic identification of METTL21A potential substrates.
(XLS)

Table S3 Summary of proteomic identification of METTL10 potential substrates.
(XLS)
