# Supplementary figures and images for: Selenium-Based S-Adenosylmethionine Analog Reveals the Mammalian Seven-Beta-Strand Methyltransferase METTL10 to Be an EF1A1 Lysine Methyltransferase
Source: PLoS One. 2014 Aug 21;9(8):e105394. doi: 10.1371/journal.pone.0105394 (PMC4140779; doi:10.1371/journal.pone.0105394)

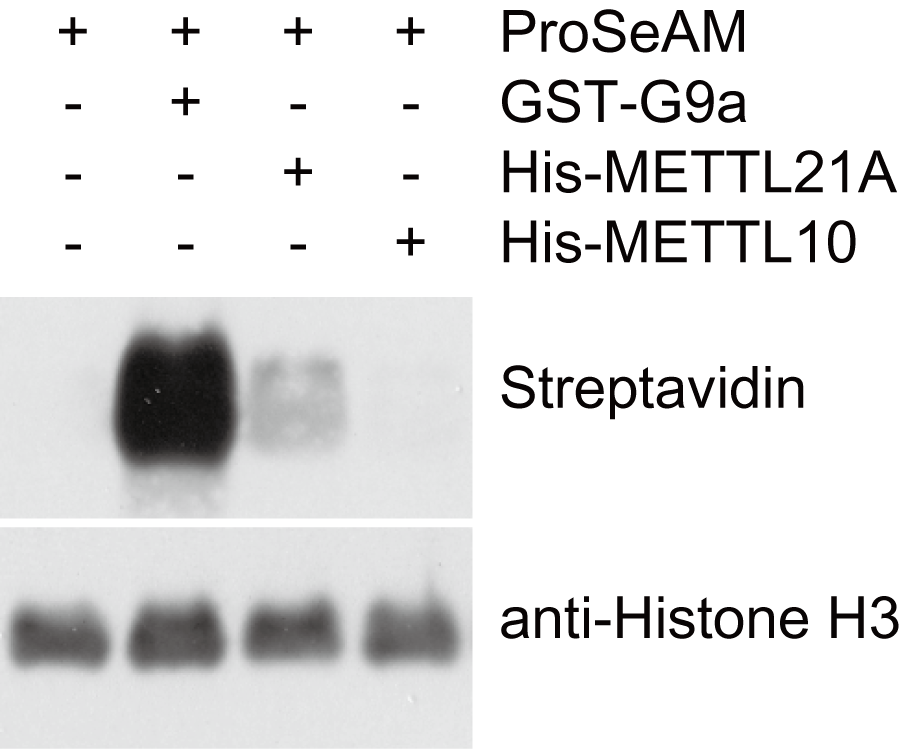

Supplement: Figure S1 — Histone H3 is labeled by METTL21A with ProSeAM. Full-length Histone H3 (1 µg) and ProSeAM (500 µM) were incubated with indicated KMTs (0.5 µg) for 2 h at 20°C. The histones were separated by SDS-PAGE, transferred to a nitrocellulose membrane and probed with streptavidin-HRP (top) or anti-Histone H3 antibody (bottom). (TIF) [file pone.0105394.s001.tif]
